# Safety, Tolerability, and Pharmacokinetics of MEDI4893, an Investigational, Extended-Half-Life, Anti-Staphylococcus aureus Alpha-Toxin Human Monoclonal Antibody, in Healthy Adults

**DOI:** 10.1128/AAC.01020-16

**Published:** 2016-12-27

**Authors:** Xiang-Qing Yu, Gabriel J. Robbie, Yuling Wu, Mark T. Esser, Kathryn Jensen, Howard I. Schwartz, Terramika Bellamy, Martha Hernandez-Illas, Hasan S. Jafri

**Affiliations:** a MedImmune LLC, Gaithersburg, Maryland, USA; b Miami Research Associates, Miami, Florida, USA

**Keywords:** MEDI4893, anti-infective monoclonal antibodies, alpha-toxin, antimicrobial safety, clinical pharmacokinetics, efficacy, nosocomial pneumonia

## Abstract

MEDI4893 is an investigational immunoglobulin G1(κ) monoclonal antibody that specifically binds to and neutralizes alpha-toxin, a key Staphylococcus aureus virulence factor. A triple-amino-acid substitution, M252Y/S254T/T256E, was engineered into the MEDI4893 Fc region to extend its serum half-life. A phase 1, double-blind, dose escalation study was designed to evaluate the safety, tolerability, pharmacokinetics, anti-alpha-toxin-neutralizing activity, and antidrug antibody (ADA) response of MEDI4893 following a single intravenous infusion in healthy adults 18 to 65 years of age. Thirty-three subjects were randomly assigned to receive MEDI4893 at 225 mg (*n* = 3), 750 mg (*n* = 3), 2,250 mg (*n* = 8), or 5,000 mg (*n* = 12) or placebo (*n* = 7) and were followed for 360 days. Adverse events were mild or moderate in severity; none were serious. The MEDI4893 peak serum concentration increased dose proportionally from 77.2 μg/ml (225-mg dose) to 1,784 μg/ml (5,000-mg dose). The area under the concentration-time curve from 0 to 360 days also increased dose proportionally, from 4,840 μg · day/ml (225-mg dose) to 91,493 μg · day/ml (5,000-mg dose), indicating linear pharmacokinetics. MEDI4893's terminal half-life was estimated to be 80 to 112 days, which is approximately 4-fold longer than the half-lives of other human immunoglobulin G antibodies. The alpha-toxin-neutralizing activity in serum correlated highly with the MEDI4893 concentrations in serum. Three adults transiently tested positive for ADA on day 151, but this did not have an impact on MEDI4893 serum concentrations or the MEDI4893 safety profile; no subjects exhibited serum ADA at the study end. These data support the continued development of MEDI4893 for the prevention of S. aureus-mediated pneumonia. (This study has been registered at ClinicalTrials.gov under identifier NCT02296320.)

## INTRODUCTION

Staphylococcus aureus is a Gram-positive coccus that causes serious infections of multiple organs, including the skin, soft tissues, respiratory tract, bone, joints, and endovascular system (1). S. aureus is the leading cause of hospital-acquired (nosocomial) pneumonia, including ventilator-associated pneumonia (2), resulting in significant morbidity, health care resource utilization, and death (3). Although antibiotics are the standard of care for S. aureus pneumonia (4, 5), treatment is complicated by increasing rates of antibiotic resistance among clinical isolates. Antibiotic-resistant S. aureus has been associated with increased rates of morbidity and mortality and an increased cost of treatment (6). These challenges warrant consideration of new approaches to the management and prevention of serious S. aureus infection.

An innovative approach to the prevention of S. aureus pneumonia could be the use of an anti-infective monoclonal antibody for immunoprophylaxis that targets a specific common virulence factor protein on S. aureus (7). Recent research has shown the S. aureus alpha-toxin protein (also called alpha-hemolysin) to be a viable target for this type of disease prevention strategy (8–12). Alpha-toxin is a highly conserved, key virulence factor of S. aureus that functions as a cytolytic pore-forming toxin that, when released into the infected host, leads to tissue disruption, programmed cell death of leukocytes and endothelial cells, bacterial dissemination, and immune dysregulation (8, 13–17). Thus, the neutralization of alpha-toxin should prevent the physiological damage caused by the toxin and limit the dissemination of S. aureus. This theory is supported by the observation that hospital isolates of S. aureus possessing a defective alpha-toxin gene have reduced virulence in mouse infection models (18).

MEDI4893 is an investigational human immunoglobulin G1(κ) [IgG1(κ)] monoclonal antibody that binds with a high affinity to and neutralizes S. aureus alpha-toxin, thereby diminishing S. aureus disease pathogenesis, as demonstrated in animal models of lethal pneumonia (8, 19). This monoclonal antibody recognizes a highly conserved region of alpha-toxin that has been identified in >97% of S. aureus clinical isolates sequenced to date around the world (17, 20) and exerts its neutralizing activity through a dual mechanism: (i) it sterically blocks binding of alpha-toxin to the toxin's cellular receptor, and (ii) it prevents alpha-toxin from adopting the pore-forming heptameric transmembrane conformation that is required for host cell lysis (19). MEDI4893 was derived from a previously described anti-alpha-toxin monoclonal antibody, LC10, and possesses a triple-amino-acid substitution (M252Y/S254T/T256E [YTE]) in the antibody Fc region that confers an extended serum half-life by increasing the affinity of antibody binding to the neonatal Fc receptor involved in lysosomal recycling of IgG molecules (21). Importantly, the YTE substitution does not interfere with the specificity of binding of antibody molecules to their target epitopes, as is evident in the ability of MEDI4893 to neutralize alpha-toxin by binding to the epitope involved in cell attachment and lytic pore formation (8, 19, 21).

MEDI4893 is currently under clinical investigation to assess its safety and efficacy in preventing S. aureus pneumonia in hospitalized, S. aureus-colonized patients (ClinicalTrials.gov identifier NCT02296320; EudraCT identifier 2014-001097-34). Described here are the results of the first-in-human dose escalation study designed to evaluate the safety, tolerability, single-dose pharmacokinetics, *ex vivo* alpha-toxin-neutralizing activity, and antidrug antibody (ADA) responses of MEDI4893 in healthy adult volunteers. The selection of MEDI4893 doses was based on good laboratory practice toxicology and human pharmacokinetic simulations performed in cynomolgus monkeys, observed and predicted efficacy from mouse pharmacology studies, and U.S. Food and Drug Administration guidelines (22). The selected doses were anticipated to deliver a range of MEDI4893 serum concentrations that would reach and maintain a protective therapeutic level above the 90% effective concentration (EC_90_) of 211 μg/ml over 30 days, which has been established in a mouse model of S. aureus pneumonia (8).

## RESULTS

A total of 33 enrolled subjects received MEDI4893 or placebo: 7, 3, 3, 8, and 12 subjects received either placebo or MEDI4893 at 225 mg, 750 mg, 2,250 mg, or 5,000 mg, respectively. In all, 30 subjects were followed through 360 days postdosing (day 361). One subject randomized to placebo was lost to follow-up (day 160), and two subjects randomized to 5,000 mg of MEDI4893 withdrew consent (on days 76 and 226, respectively) for reasons other than adverse events. Demographic data were fairly balanced across all cohorts (Table 1).

**TABLE 1:** Subject demographics and baseline characteristics^*a*^

Characteristic	Value for:				
	Cohort 1 (*n* = 4)	Cohort 2 (*n* = 4)	Cohort 3 (*n* = 10)	Cohort 4 (*n* = 15)	All subjects (*n* = 33)
No. (%) of subjects by sex					
Male	2 (50)	2 (50)	6 (60)	7 (47)	17 (52)
Female	2 (50)	2 (50)	4 (40)	8 (53)	16 (48)
No. (%) of subjects by race					
White	3 (75)	4 (100)	9 (90)	13 (87)	29 (88)
Black or African American	1 (25)	0	1 (10)	2 (13)	4 (12)
Mean ± SD age (yr)	39.5 ± 12.8	37.8 ± 16.5	33.8 ± 8.7	42.9 ± 8.5	39.1 ± 10.5
Mean ± SD body wt (kg)	84.0 ± 18.0	77.9 ± 12.2	73.9 ± 14.9	80.6 ± 9.7	78.7 ± 12.7

a Cohort 1 received MEDI4893 at 225 mg or placebo, cohort 2 received MEDI4893 at 750 mg or placebo, cohort 3 received MEDI4893 at 2,250 mg or placebo, and cohort 4 received MEDI4893 at 5,000 mg or placebo.

One or more adverse events were reported in 11 of the 26 subjects (42.3%) who received MEDI4893 and 4 of the 7 subjects (57.1%) who received placebo (Table 2). All adverse events were mild or moderate in severity. Four subjects had adverse events that were considered to be related to the study drug, as determined by the principal investigator. These adverse events included (i) headache that lasted for 1 to 2 days and that was reported to be mild in severity in two subjects (one receiving 225 mg of MEDI4893 and the other receiving 2,250 mg of MEDI4893), (ii) urticaria that lasted for 4 days and that was moderate in severity in one subject who received 2,250 mg MEDI4893, and (iii) rash that was mild in severity and that lasted for 2 days in one subject who received 5,000 mg of MEDI4893. There were no serious adverse events, deaths, or study drug discontinuations attributed to adverse events through the end of the study.

**TABLE 2:** Adverse events in subjects receiving MEDI4893 or placebo^*a*^

MEDI4893 dose or placebo (no. of subjects)	No. (%) of adverse events by system organ class							
	All subjects reporting ≥1 AE	Upper respiratory tract infection	Headache	Rash	Urticaria	GERD	Vomiting	Infusion site extravasation
Placebo (*n* = 7)	4 (57.1)	1 (14.3)	2 (28.6)	0 (0)	0 (0)	0 (0)	0 (0)	1 (14.3)
225 mg (*n* = 3)	2 (66.7)	2 (66.7)	1 (33.3)	0 (0)	0 (0)	0 (0)	0 (0)	0 (0)
750 mg (*n* = 3)	2 (66.7)	1 (33.3)	0 (0)	0 (0)	0 (0)	1 (33.3)	0 (0)	0 (0)
2,250 mg (*n* = 8)	3 (37.5)	0 (0)	1 (12.5)	0 (0)	1 (12.5)	0 (0)	0 (0)	0 (0)
5,000 mg (*n* = 12)	4 (33.3)	1 (8.3)	1 (8.3)	1 (8.3)	0 (0)	0 (0)	1 (8.3)	0 (0)

a No serious adverse events were reported by any study subjects. Abbreviations: AE, adverse event; GERD, gastroesophageal reflux disease.

**FIG 1:**
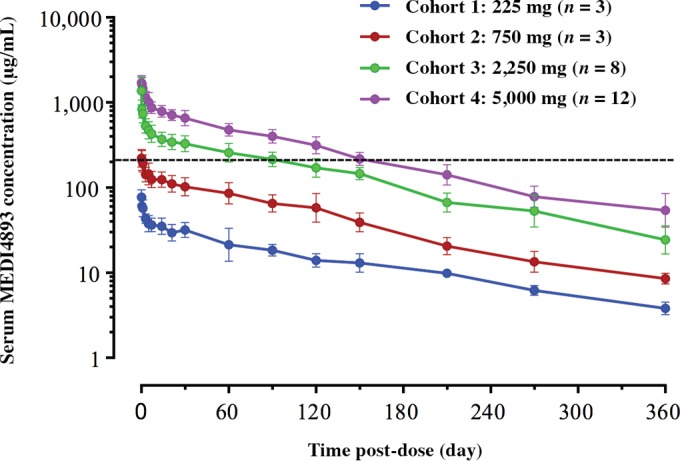
MEDI4893 pharmacokinetics in healthy adult volunteers. Dashed line, the target concentration of 211 μg/ml, which is the EC_90_ from a S. aureus murine pneumonia model.

MEDI4893 exhibited linear pharmacokinetics with low intersubject variability (Fig. 1). Serum exposure of MEDI4893 increased approximately dose proportionally; the maximum concentration of drug in serum (*C*_max_) increased from 77 μg/ml at 225 mg to 1,784 μg/ml at 5,000 mg (Table 3). The mean serum area under the concentration-time curve (AUC) from 0 to 360 days postdosing (AUC_0–360_) increased from 4,840 μg · day/ml at 225 mg to 91,496 μg · day/ml at 5,000 mg. The clearance of MEDI4893 from serum ranged from 42 ml/day to 50 ml/day, which is about 4-fold lower than that of humanized, chimeric, or human IgG1 monoclonal antibodies (23). The terminal half-life of MEDI4893 was estimated to be 80 to 112 days. MEDI4893 serum levels were greater than the therapeutic target concentration of 211 μg/ml (informed by the EC_90_ in the mouse pneumonia model) for at least 60 days in the 2,250-mg-dose group and at least 120 days in the 5,000-mg-dose group (Fig. 1), thereby exceeding the anticipated protective level of MEDI4893 over those extended time periods postinfusion. The volume of distribution of MEDI4893 at steady state ranged from 4.9 liters to 6.6 liters, indicating a limited distribution to the extravascular space (Table 3).

**TABLE 3:** MEDI4893 serum pharmacokinetic parameters for each dose determined by noncompartmental analysis^*a*^

MEDI4893 dose (no. of subjects)	*C*_max_ (μg/ml)	AUC_0–360_ (μg · day/ml)	AUC_0–∞_ (μg · day/ml)	Clearance (ml/day)	*V*_ss_ (ml)	Half-life (days)
225 mg (*n* = 3)	77 ± 17	4,840 ± 992	5,457 ± 1,002	42 ± 9	6,611 ± 1,973	112 ± 20
750 mg (*n* = 3)	221 ± 60	15,869 ± 4,677	16,897 ± 4,521	47 ± 14	5,687 ± 2,021	83 ± 5
2,250 mg (*n* = 8)	1,397 ± 613	50,780 ± 10,746	53,892 ± 12,388	44 ± 10	4,921 ± 849	84 ± 13
5,000 mg (*n* = 12)	1,784 ± 396	91,496 ± 25,463	10,4437 ± 22,709	50 ± 11	5,450 ± 793	80 ± 12

a Data are expressed as the mean ± standard deviation. Abbreviation: *V*_ss_, volume of distribution at steady state.

Measurable levels of MEDI4893 were also detected in nasal wash samples at all dose levels, but the intersubject and intrasubject variabilities over time were high. The mean MEDI4893 *C*_max_ in nasal wash samples increased with dose from 39 ng/ml at 225 mg to 538 ng/ml at 5,000 mg, and the mean AUC from time zero to 30 days postdosing (AUC_0–30_) in nasal wash samples increased from 288.3 ng · day/ml at 225 mg to 6,314.3 ng · day/ml at 5,000 mg (Table 4). Similarly, the mean AUC from time zero to the time of the last quantifiable concentration (AUC_last_) of MEDI4893 in nasal wash samples also increased with dose from 287.5 ng · day/ml at 225 mg to 20,407.6 ng · day/ml at 5,000 mg. MEDI4893 was detected in nasal wash samples up to day 31 postinfusion in 2 of 3 subjects in the 225-mg dose cohort; up to day 121 postinfusion in the majority (≥67%) of subjects in the 750-mg, 2,250-mg, and 5,000-mg dose cohorts; and up to day 151 in 10 of 12 (83%) subjects in the 5,000-mg dose cohort (data not shown).

**TABLE 4:** MEDI4893 nasal pharmacokinetic parameters for each dose determined by noncompartmental analysis^*a*^

MEDI4893 dose (no. of subjects)	*C*_max_ (ng/ml)	AUC_0–30_ (ng · day/ml)	AUC_last_ (ng · day/ml)	*C*_max_ coefficient (nasal/serum)	AUC_0–30_ coefficient (nasal/serum)
225 mg (*n* = 3)	39.1 ± 23.9	288.3 ± 9.4	287.5 ± 225.9	0.00048 ± 0.00018	0.00031 ± 0.00002
750 mg (*n* = 3)	144 ± 126	3,237.1 ± 2,367.0	5,135.0 ± 6,078.5	0.00057 ± 0.00047	0.00074 ± 0.00046
2,250 mg (*n* = 8)	211.7 ± 196.5	2,103.3 ± 1,716.4	8,275.8 ± 8,377.9	0.00023 ± 0.00030	0.00019 ± 0.00017
5,000 mg (*n* = 12)	537.6 ± 447.1	6,314.3 ± 4,586.1	20,407.6 ± 20,126.5	0.00031 ± 0.00029	0.00026 ± 0.00021

a Data are expressed as the mean ± standard deviation.

**FIG 2:**
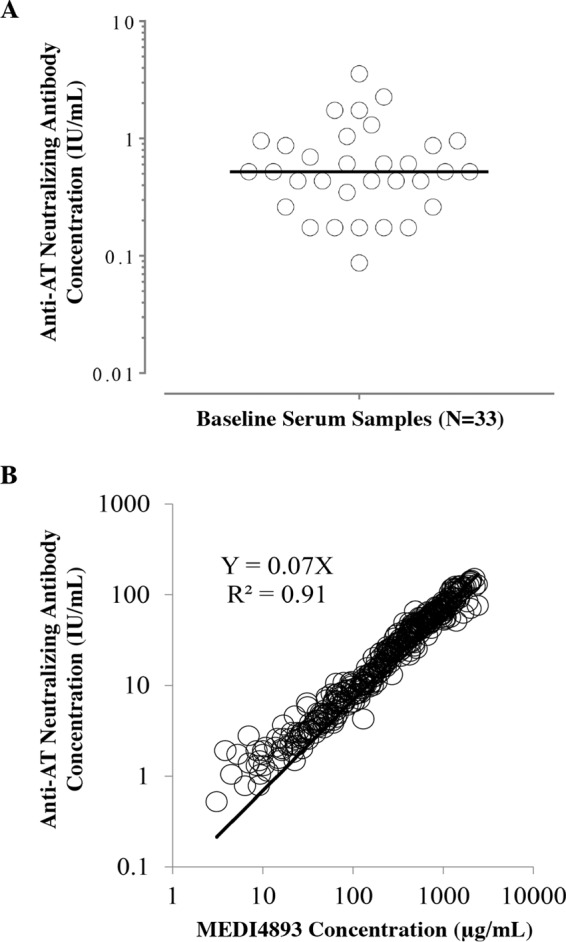
(A) Baseline serum anti-alpha-toxin-neutralizing antibody concentration. (B) Correlation between serum MEDI4893 concentrations and anti-alpha-toxin-neutralizing concentration. AT, alpha-toxin.

Individual baseline serum concentrations of anti-alpha-toxin-neutralizing antibody in the study participants were low, ranging from 0.1 IU/ml to 3.6 IU/ml, and the median concentration was 0.52 IU/ml (Fig. 2A). The serum concentrations of anti-alpha-toxin-neutralizing antibody increased in a dose-dependent manner after MEDI4893 dosing and were highly correlated with the serum concentrations of MEDI4893 across the cohorts at all time points (Fig. 2B). The median levels of serum anti-alpha-toxin-neutralizing antibody at the end of MEDI4893 infusion (day 1) were 7-, 36-, 247-, and 238-fold greater than the median preinfusion baseline levels in the 225-, 750-, 2,250-, and 5,000-mg, dose cohorts, respectively. The serum levels of anti-alpha-toxin-neutralizing antibody were sustained through 30 days postinfusion (day 31) and were 3-, 15-, 91-, and 92-fold higher than the median preinfusion baseline levels in the 225-, 750-, 2,250-, and 5,000-mg dose cohorts, respectively. The serum levels of anti-alpha-toxin-neutralizing antibody in the 750-, 2,250-, and 5,000-mg dose cohorts at day 31 were >3.2 IU/ml, a concentration that has been found to be associated with favorable clinical outcomes in epidemiology studies (20, 24). These data confirm the *ex vivo* alpha-toxin-neutralizing activity of MEDI4893 and show that high levels can be maintained through 30 days after intravenous (i.v.) administration.

Four subjects tested positive for MEDI4893 ADA. One subject who received 2,250 mg of MEDI4893 tested positive at the baseline, before administration of the study drug; two subjects, one who received 2,250 mg and one who received 5,000 mg, tested positive both at the baseline and on day 151; and one subject who received 5,000 mg tested positive on day 151 only. All four subjects had a low ADA titer (≤1:30), and none of the subjects tested positive for ADA at the end of the study. The transient presence of ADA was not associated with any adverse events or any impact on MEDI4893 pharmacokinetics.

## DISCUSSION

The MEDI4893 molecule is being developed to provide S. aureus disease prevention for mechanically ventilated patients who are at risk for nosocomial pneumonia. Data from preclinical studies suggest that MEDI4893 would provide effective immunoprophylaxis against S. aureus nosocomial pneumonia.

In this phase 1, first-in-human, dose escalation, placebo-controlled trial, administration of MEDI4893 was not associated with any serious adverse events, deaths, or study discontinuations throughout the study period. Only four adverse events (two cases of headaches, one case of urticaria, and one case of rash) were deemed to be potentially related to the study drug and were transient and mild in severity, supporting the overall acceptable safety profile of MEDI4893.

MEDI4893 exhibited linear pharmacokinetics, showing an extended half-life in healthy adults. The observed MEDI4893 half-life of 80 to 112 days is approximately 4-fold longer than that of other human IgG1 monoclonal antibodies, confirming the antibody half-life extension in humans conferred by the YTE substitution (25–28). The pharmacokinetic profiles of MEDI4893 are similar to those of another antibody engineered to have the YTE substitution, motavizumab-YTE, an anti-respiratory syncytial virus IgG1 monoclonal antibody. Motavizumab-YTE showed an extended half-life of 70 to 100 days in healthy adults, which is also approximately 4-fold longer than that of wild-type motavizumab (29). In addition, the extension of the half-life of MEDI4893 by YTE engineering in humans is consistent with that observed in cynomolgus monkeys, in which the terminal half-life of MEDI4893 was estimated to be approximately 2- to 4-fold longer than that of regular IgG1 antibodies (30). The consistency of the pharmacokinetics across the two species suggests that, like regular IgG1 antibodies, the pharmacokinetics of the MEDI4893 antibody with YTE in cynomolgus monkeys can be predictive of those in humans.

Following a single i.v. infusion, MEDI4893 was distributed to the upper respiratory tract, which is relevant, given the intended target disease of S. aureus nosocomial pneumonia. Despite the high individual and intergroup variability in the levels of MEDI4893 in nasal wash samples, the data presented here demonstrated trends for dose-dependent increases and time-dependent decreases in MEDI4893 levels in nasal wash samples, consistent with the pharmacokinetics in serum. An ongoing phase 2 trial (ClinicalTrials.gov identifier NCT02296320) is currently evaluating the distribution of MEDI4893 into the upper airway and its safety and efficacy as immunoprophylaxis against nosocomial pneumonia in mechanically ventilated patients colonized with S. aureus.

At present, limited data on the immunogenicity of monoclonal antibodies with the YTE substitution are available. A pharmacokinetic and safety study of the anti-respiratory syncytial virus monoclonal antibody motavizumab-YTE demonstrated comparable incidences of detectable ADA responses in healthy subjects receiving single i.v. infusions of motavizumab-YTE (4 of 16 subjects; 25%) and in subjects receiving the nonsubstituted antibody (3 of 15 subjects; 20%) (29). In that study, the presence of ADA did not affect the pharmacokinetics of motavizumab-YTE but did increase the clearance of nonsubstituted motavizumab in two of three ADA-positive subjects (29). In the current study, both the frequency of occurrence and the level of serum ADA were low and transient; three of four subjects who received MEDI4893 had low titers of ADA at the baseline, and none of the subjects tested positive for ADA at the end of the study. Transient serum ADA responses were not associated with any impact on MEDI4893 safety or serum pharmacokinetics.

The *ex vivo* anti-alpha-toxin-neutralizing antibody assay utilized in this study provides a sensitive and specific measure of MEDI4893's capacity to effectively neutralize alpha-toxin as a surrogate indicator of the antibody's potential efficacy *in vivo*. This assay has also previously been used to map the epitope on alpha-toxin recognized by MEDI4893, identified to be the portion of the alpha-toxin rim domain involved in host cell attachment (19).

In this study, the *ex vivo* alpha-toxin-neutralizing activity of MEDI4893 in serum increased substantially (75- to 238-fold) after MEDI4893 dosing and showed a high correlation with MEDI4893 exposure across all dose levels, indicating the intact functionality of MEDI4893 in humans. In addition, the alpha-toxin-neutralizing capacity of MEDI4893 was maintained through 30 days postdosing, during which the concentration remained above 3.2 IU/ml, a threshold that has been shown in a previous epidemiology study to correlate with a reduced incidence of S. aureus infections. These results confirm that the YTE amino acid substitution does not diminish the binding or alpha-toxin-neutralizing characteristics of MEDI4893, while it extends its serum pharmacokinetic profile.

Overall, the safety, pharmacokinetic, ADA, and pharmacodynamic data from this first-in-human study of MEDI4893 support the development of MEDI4983 to provide sustained passive immunity against S. aureus disease.

## MATERIALS AND METHODS

### Study objectives and design.

This first-in-human, double-blind, randomized, placebo-controlled, dose escalation, phase 1 study was conducted at a single study center in the United States. The primary objective of the study was to evaluate the safety and tolerability of a single i.v. dose of MEDI4893 administered to healthy adults. The secondary objectives were to evaluate the pharmacokinetics of MEDI4893 and to measure ADA levels in serum. Exploratory objectives included measurement of MEDI4893 levels in the upper respiratory tracts of subjects and anti-alpha-toxin-neutralizing antibody concentrations in serum and the upper respiratory tract before and after administration of MEDI4893.

Subjects were randomized to one of four fixed-dose cohorts as follows: a 3:1 ratio of subjects receiving MEDI4893 to subjects receiving placebo in cohorts 1 (225 mg) and 2 (750 mg) and a 4:1 ratio of subjects receiving MEDI4893 to subjects receiving placebo in cohorts 3 (2,250 mg) and 4 (5,000 mg). To review safety parameters and accommodate dose escalation decisions, dosing was staggered such that the first two subjects in cohort 1 were randomized to MEDI4893 or placebo, dosed at least 48 h apart, and followed for 7 days postdosing before the remaining two subjects in cohort 1 were dosed (the remaining two subjects were also dosed at least 48 h apart). If no safety concerns were observed in these four subjects, additional subjects were enrolled in the cohort receiving the next-higher dose. Randomization to subsequent dose levels continued once the 7-day observation period after administration of the previous dose had elapsed.

The study duration included a screening period of up to 28 days and a 360-day pharmacokinetic and safety evaluation follow-up period. Subject visits were every 2 days for the first week (days 2, 4, 6, and 8) and then every week until day 31 (days 15, 22, and 31), every month for the following 4 months (days 61, 91, 121, and 151), and every 2 months until the study end (days 211, 271, and 361).

### Inclusion and exclusion criteria of subjects.

Healthy adult volunteers 18 to 65 years of age were eligible to participate in the study. Subjects had to be healthy on the basis of their medical history and physical examination at screening and had to weigh 45 to 110 kg. Subjects also had to have a systolic blood pressure of <140 mm Hg, a diastolic blood pressure of <90 mm Hg, and a normal electrocardiogram at screening. Subjects who had previously received monoclonal antibodies were excluded, as were those who had received immunoglobulin or blood products within the 6 months before study entry or any investigational drug or investigational vaccine within the 120 days before dosing with the study drug.

Written informed consent was obtained from subjects before study procedures began. The Institutional Review Board (Aspire IRB, Santee, CA, USA) approved the study protocol and informed consent documents. The study was conducted in accordance with the ethical principles of the Declaration of Helsinki and the International Council for Harmonization guidelines on good clinical practice.

### Assessments.

Enrolled subjects were admitted to a phase 1 study unit for pre- and postdosing observation. Vital signs were assessed predosing; every 30 min during study drug infusion; at the completion of the infusion; and at 30 min, 60 min, and 2, 6, 8, and 24 h after the completion of study drug infusion.

Safety assessments included recording of adverse events that occurred during the period immediately after study drug dosing through 90 days postdosing, as well as reporting of serious adverse events, new-onset chronic diseases, and adverse events of special interest (hepatic function abnormality; anaphylaxis, hypersensitivity, and infusion reactions; immune complex disease) that occurred through 360 days postdosing.

Serum samples were collected at the baseline (predose on day 1) and at 1 h and 8 h postdosing on day 1 and during study visits on days 2, 4, 6, 8, 15, 22, 31, 61, 91, 121, 151, 211, 271, and 361 postdosing to evaluate the systemic pharmacokinetic profile of MEDI4893 and the levels of anti-alpha-toxin-neutralizing antibody. Nasal wash samples were also collected at the same times that serum samples were collected (except on day 6) for analysis of the pharmacokinetics of MEDI4893 in the upper respiratory tract. MEDI4893 ADA levels in serum samples collected at the baseline and on days 15, 31, 91, 151, 211, 271, and 361 were measured.

### Pharmacokinetic analysis of MEDI4893.

The levels of MEDI4893 in both serum and nasal wash samples were measured using a validated enzyme-linked immunosorbent assay. Briefly, an immobilized anti-YTE monoclonal antibody was used to capture MEDI4893 in human serum samples and nasal washes, and a second biotinylated anti-MEDI4893 monoclonal antibody was used for MEDI4893 detection. The MEDI4893 concentration was estimated by interpolating the observed value from a 1/*y*^2^-weighted, four-parameter logistic function, standard curve fit that was generated using SoftMax Pro software (v5.2; Molecular Devices Corporation, Sunnyvale, CA). The lower and upper limits of quantitation were 0.23 μg/ml and 15 μg/ml, respectively, for serum samples and 3.5 ng/ml and 100 ng/ml, respectively, for nasal wash samples.

Noncompartmental pharmacokinetic analysis was performed using Phoenix WinNonlin software (Pharsight, CA). The peak (maximum) concentration in serum (*C*_max_) of MEDI4893 was defined as the observed individual maximum concentration after dosing. The terminal elimination half-life was determined using a log-linear regression of the concentration data with the equation ln(2)/λ_*z*_, where λ_*z*_ is the slope of the terminal portion of the natural log concentration-time curve, determined by linear regression of the data from at least the last three time points. The systemic exposure was determined by calculating the area under the serum concentration-time curve (AUC) from 0 to 360 days postdosing (AUC_0–360_), using a linear-log trapezoidal method. The serum AUC of MEDI4893 from time zero to infinity (AUC_0–∞_) was calculated as AUC_last_ + *C*_last_/λ_*z*_, where AUC_last_ is the area under the serum concentration-time curve from time zero to the time of the last quantifiable concentration and *C*_last_ is the last quantifiable concentration in serum.

### Anti-alpha-toxin-neutralizing antibodies.

Serum anti-alpha-toxin-neutralizing antibody levels were measured with a red blood cell-based neutralization assay, utilizing the National Institute for Biological Standards and Control's World Health Organization international reference standard (31). The lower limit of quantitation was 0.0007 international unit (IU) per ml, and anti-alpha-toxin-neutralizing antibody levels were reported in international units per milliliter, as described previously (20).

### Anti-MEDI4893 antibodies.

A validated electrochemiluminescent, solution-phase, bridging immunoassay was used for the detection, confirmation, and titration of anti-MEDI4893 antibodies (ADA) in human serum. Briefly, a mixture of biotinylated and ruthenylated MEDI4893 was used to capture the ADA present in serum samples. The ADA-bridged immune complexes were then bound to a streptavidin-coated mesoscale discovery plate. Samples with electrochemiluminescent responses equal to or above the cutoff point value (1.37 times the mean response for six to eight wells of the negative control in each plate) were considered positive. Positive detection of anti-MEDI4893 antibodies was defined as a titer of ≥1:30.
